# BCL2L10 positive cells in bone marrow are an independent prognostic factor of azacitidine outcome in myelodysplastic syndrome and acute myeloid leukemia

**DOI:** 10.18632/oncotarget.17482

**Published:** 2017-04-27

**Authors:** Valérie Vidal, Guillaume Robert, Laure Goursaud, Laetitia Durand, Clemence Ginet, Jean Michel Karsenti, Frederic Luciano, Lauris Gastaud, Georges Garnier, Thorsten Braun, Pierre Hirsch, Emmanuel Raffoux, Anne Marie Nloga, Rose Ann Padua, Hervé Dombret, Pierre Rohrlich, Lionel Ades, Christine Chomienne, Patrick Auberger, Pierre Fenaux, Thomas Cluzeau

**Affiliations:** ^1^ INSERM U1131, Institut Universitaire d’hématologie, Paris, France; ^2^ INSERM U1065, Centre Méditerranéen de Médecine Moléculaire, Nice, France; ^3^ Cote d'azur University, Nice Sophia Antipolis University, CHU of Nice, Nice, France; ^4^ Centre Antoine Lacassagne, Service d’oncologie, Nice, France; ^5^ CH Princesse Grace, Service de Médecine Interne, Monaco, Monaco; ^6^ Hôpital Avicenne, Paris 13 University, APHP, Bobigny, France; ^7^ Hôpital Saint Antoine, Service d'Hématologie Clinique et de Thérapie Cellulaire, Paris, France; ^8^ Sorbonne Universités, UPMC Univ Paris 6, UMRS 938, CDR Saint-Antoine, Paris, France; ^9^ Hôpital Saint Louis, Paris 7 University, Service d’Hématologie Adulte, APHP, Paris, France; ^10^ Hôpital Saint Louis, Paris 7 University, Service d’Hématologie Sénior, APHP, Paris, France

**Keywords:** MDS, AML, BCL2L10, IPSS, IPSS-R

## Abstract

Azacitidine (AZA), the reference treatment for most higher-risk myelodysplastic (MDS) patients can also improve overall survival (OS) in elderly acute myeloid leukemia (AML) patients ineligible for intensive chemotherapy, but reliable biological markers predicting response and OS in patients treated with AZA are lacking. In a preliminary study, we found that an increase of the percentage of BCL2L10, an anti-apoptotic member of the bcl-2 family, was correlated with AZA resistance. In this study, we assessed prospectively by flow cytometry the prognostic value of BCL2L10 positive bone marrow mononuclear cells in 70 patients (42 MDS and 28 AML), prior to AZA treatment.

In patients with baseline marrow blasts below 30%, the baseline percentage of bone marrow BCL2L10 positive cells inversely correlated with response to AZA and OS independently of the International Prognostic Scoring System (IPSS) and IPSS-revised (IPSS-R). Specifically, OS was significantly lower in patients with more than 10% BCL2L10 positive cells (median 8.3 vs 22.9 months in patients with less than 10% positivity, *p* = 0,001). In summary, marrow BCL2L10 positive cells may be a biomarker for azacitidine response and OS, with a potential impact in clinical practice.

## INTRODUCTION

Azacitidine (AZA) is the reference treatment for most higher-risk myelodysplastic syndromes (MDS) patients [1] which also improves overall survival (OS) in elderly acute myeloid leukemia (AML) patients ineligible for intensive chemotherapy (IC) [2]. In MDS, conventional biological factors included in the international prognosis scoring system (IPSS) are BM blasts percentage, karyotype and number of cytopenias. In the revised IPSS (IPSS-R), BM blasts percentage, cytogenetic risk group, hemoglobin, platelets and absolute neutrophil counts are used as parameters. The French Prognostic Scoring System (FPSS) is based on ECOG, presence of circulating blasts, red blood cell transfusion dependence and cytogenetic risk group. These scores can predict response and OS to AZA treatment [3–6]. Several genomic alterations detected by single nucleotide polymorphism (SNP) array and methylation profiles-such as *TET2* mutations-have also been reported to predict better response to hypomethylating agents (HMA). Nevertheless, most of those results were not reproducible, did not allow to predict survival or were hardly applicable in routine practice [7–11]. An exception is the consistently poor prognosis associated with *TP53* mutation, but this mutation is largely correlated with the presence of a complex karyotype [12].

BCL2L10 is an antiapoptotic [13–15] member of the bcl-2 family known to play a role in the chemoresistance of various cancers [16]. Overexpression of BCL2L10 has been reported to suppress apoptosis through inhibition of cytochrome c release from mitochondria [14]. The BCL2L10 protein undergoes several steps of regulation including final ubiquitinylation [17], confirming the interest to quantify this protein by flow cytometry (FCM) rather than to explore gene expression only [18]. In our retrospective study in 77 MDS patients evaluated at various time points after AZA onset (but generally not before AZA), we established by FCM that a high percentage of BCL2L10 positive bone marrow mononuclear cells (BMMC) was significantly associated with a lower response rate and shorter survival [18]. In this context, the aim of the present study was to prospectively validate the potential of BCL2L10 expression as a predictive biomarker of AZA treatment in MDS and AML.

## RESULTS AND DISCUSSION

### Baseline patient characteristics

Seventy MDS or AML patients, with a median age of 73 years (range 35–92) and M/F 41/29, were analyzed for bone marrow (BM) cell BCL2L10 positivity before AZA treatment. WHO classification of MDS patients at onset of AZA therapy is summarized in Table 1: 37%, 13%, 22%, 17% and 11% of patients had refractory anemia or refractory cytopenia with multilineage dysplasia (RA/RCMD), refractory anemia with excess blasts type 1 (RAEB-1), refractory anemia with excess blasts type 2 (RAEB-2), AML with less than 30% blasts and AML with more than 30% blasts, respectively. Cytogenetic risk according to IPSS and IPSS-R is also summarized in Table 1. In patients with less than 30% blasts, IPSS was low, int-1, int-2 and high in 10%, 41%, 24% and 25%, respectively; and IPSS-R was very low, low, int, high and very high in 6%, 6%, 33%, 18% and 37%, respectively. Patients were treated with AZA for a median number of 4 cycles (range 1–21). 39/70 (56%) patients received 3 or more cycles of AZA.

**Table 1:** Baseline patient characteristics in 70 MDS AML patients tested before AZA onset

	*n* = 70
**Median age (range)**	73 (35–92)
**M/F**	41/29
**Median number of AZA cycles**	4 (1–13)
**WHO Classification (%)**	
RA/RCMD	37
RAEB-1	13
RAEB-2	22
AML (and AML with > 30% blasts)	28 (11)
**Cytogenetics according to IPSS (%)**	
Good	60
Intermediate	24
Poor	16
**Cytogenetics according to IPSS-R (%)**	
Very good	2
Good	57
Intermediate	22
Poor	15
Very poor	4
**IPSS (MDS and AML ≤ 30%) (%)**	
Low	10
intermediate 1	41
intermediate 2	24
High	25
**IPSS-R (MDS and AML ≤ 30% )(%)**	
very low	6
Low	6
intermediate	33
High	18
very high	37
**Response to AZA (%)**	50

### The percentage of BCL2L10 positive cells has an impact on response and OS with AZA

**Figure 1:**
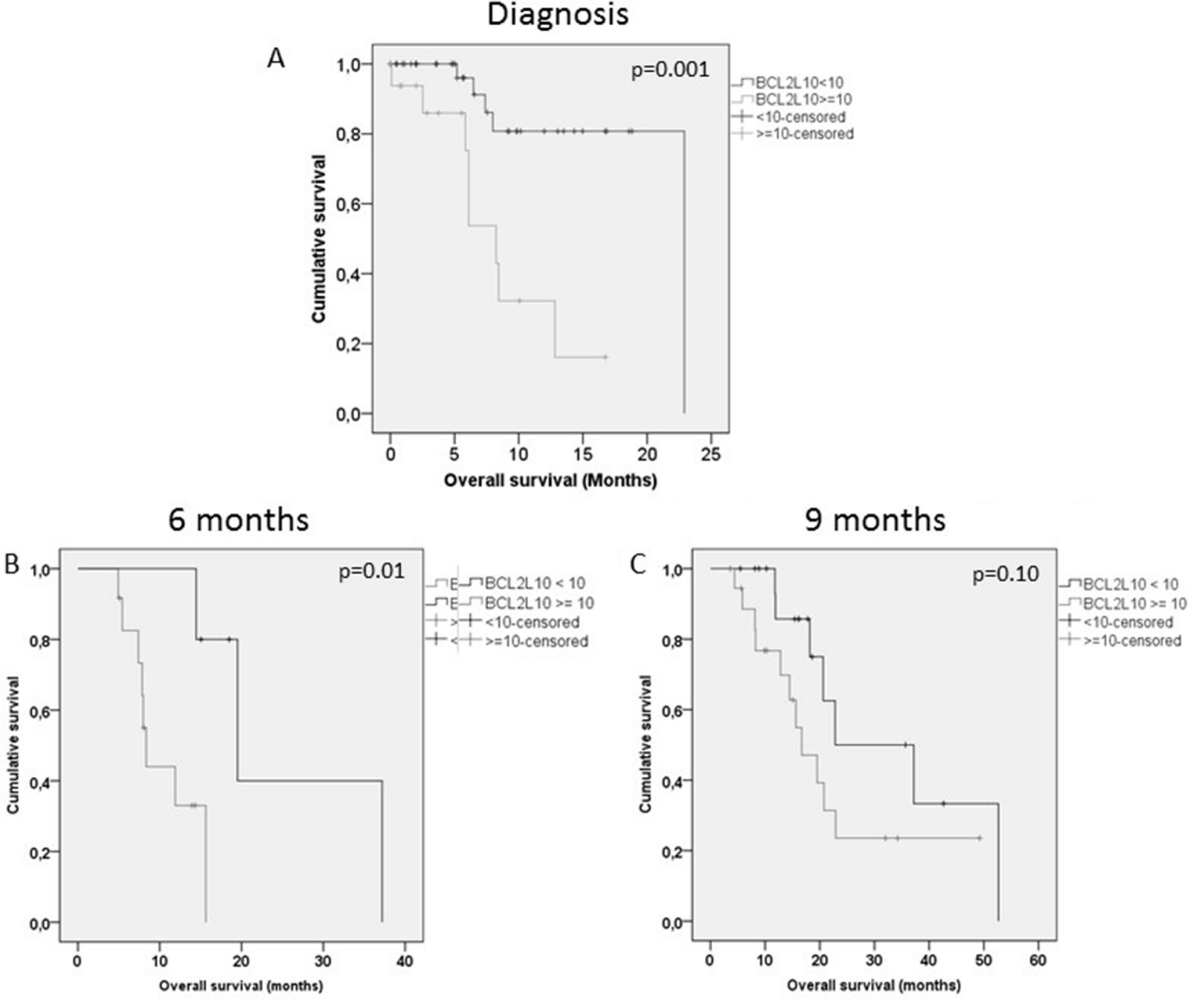
Overall survival according to BCL2L10 in patients at AZA onset, 6 months and 9 months

No significant correlation was found between BCL2L10 positive cell percentage at AZA onset and bone marrow blast percentage. Fifty percent of patients were responders after at least 3 AZA cycles including 17% of complete remission. The median baseline number of BCL2L10 positive cells was 11% (range 0–95) in AZA resistant patients vs 1% (0–41) in AZA responders (*p* = 0.09). Patient characteristics according to BCL2L10 positive cells are shown in Supplementary Table 1. We observed a trend for AZA response depending to BC2L10 (*p* = 0.08). But there is a significant difference in CR/mCR rate between patients with more than 10% versus patients with less than 10% of marrow BCL2L10 positive cells, 0% versus 50%, *p* < 0.0001. Several studies have shown CR to be most predictive of long term outcomes [21]. In addition, hypomethylating agents were often considered prior to allo stem cell transplantation in order to obtain CR. Having a biomarker tool that could predict CR and/or marrow CR could be very important to define the best cytoreductive strategy before allo stem cell transplantation. If Using a cut off value of 10%, OS was significantly lower in patients with more than 10% versus patients with less than 10% of marrow BCL2L10 positive cells: median 8.3 [5.9–10.6] vs 22.9 months [13.^8^–^22^.^9^], *p* = 0.001) (Figure 1A). An increase of BCL2L10 positive cell percentage was observed during the course of AZA treatment (median 2% [0–95] at AZA onset; 1% [0–51] after 3 cycles; 12% [0–95] after 6 cycles; 46% [28–93] after 9 cycles), and this increase was significantly greater in resistant than in sensitive patients. Increase of BCL2L10, an antiapoptic protein, could contribute to the resistance to AZA that progressively develops during AZA treatment in MDS and AML patients, explaining why most responses are transient. Using this same cutoff value, we also observed a significantly lower OS in patients with more than 10% versus less than 10% marrow BCL2L10 positive cells at 6 months and 9 months (Figure 1B and 1C).

Current results confirm prospectively, in a broader cohort of 171 MDS or AML patients, those of our first report. We also showed an increase of BCL2L10 positive cell percentage during AZA treatment, which was significantly higher in resistant than in responding patients. The median level of BCL2L10 expression was higher in AZA resistant patients (median 16%) than in responders (median 5%) (*p* = 0.005). Median survival after BCL2L10 evaluation was significantly shorter in patients with more than 10% versus patients with less than 10% BCL2L10 positive cells during AZA treatment (9 months [6–11.9] vs 15.6 months [9.6–21.7], *p* = 0.001).

### Other prognostic factors and multivariate analysis

The IPSS (HR = 1.83 [1, 11–3, 05], *p* = 0.02) and IPSS-R (HR = 2.68 [1.08–6.6], *p* = 0.03) were correlated to OS, but not the FPSS. In multivariate analysis, in MDS and AML with less than 30% of blasts, BCL2L10 positive cells percentage was predictive for OS independently of the IPSS and IPSS-R (Table 2).

**Table 2:** Uni and multivariate analysis

	*Univariate analysis*	*Multivariate analysis*
**IPSS**	HR = 1.83 (1.11–3.05), *p* = 0.02	HR = 1.57 (0.87–2.85), *p* = 0.14
**% of BCL2L10 positive cells**	HR = 2.31 (1.32–4.05), *p* = 0.004	HR = 5.46 (1.38–21.66), *p* = 0.02
	*Univariate analysis*	*Multivariate analysis*
**IPSS-R**	HR = 2.68 (1.08–6.66), *p* = 0.03	HR = 2.16 (0.83–5.62), *p* = 0.12
**% of BCL2L10 positive cells**	HR = 2.31 (1.32–4.05), *p* = 0.004	HR = 7.31 (1.52–35.25), *p* = 0.02

Therefore, in this prospective multicenter cohort, we found that the baseline percentage of BCL2L10 positive cells was inversely correlated with OS after AZA treatment in both MDS and AML patients, independently from IPSS and IPSS-R in MDS and AML with less than 30% of blasts. The best prognostic cut-off value for BCL2L10 positive cells was 10%.

We also confirmed the progressive increase in BCL2L10 expression during AZA treatment, observed in our previous retrospective series [18], with a greater increase in patients resistant to treatment.

The flow cytometry assay we used can be performed routinely in patients before the onset of AZA. Patients with more than 10% BCL2L10 bone marrow mononuclear positive cells at AZA onset, who respond poorly to single agent treatment with AZA, may require alternative therapeutic approaches, including combinations of AZA with other drugs [22–28] which are currently under investigation.

## MATERIALS AND METHODS

### Patients

MDS and AML patients, diagnosed according to WHO criteria and treated with AZA, were prospectively included in 6 centers (University hospitals of Paris - Saint Louis, Paris Saint Antoine, Paris Avicenne, University hospital of Nice, Antoine Lacassagne center (Nice) and Princesse Grace hospital (Monaco)) in a cooperative study (https://clinicaltrials.gov/: *NCT 01210274*). Patients were treated with AZA (75 mg/m^2^/day, 7 days every 4 weeks), through the drug label in IPSS int-2 or high MDS, or AML with marrow blasts less than 30%, and in compassionate use programs in AML with > 30% marrow blasts and IPSS low and int-1 MDS. Patients had bone marrow evaluation every 3 cycles. Patients without response according to IWG 2006 criteria for MDS [19] or by Cheson et al. (2003) criteria for AML [20] at 6 cycles were considered as non-responders. Responses included in MDS: complete response (CR), partial response (PR), marrow CR (mCR), and hematologic improvement (HI). For AML, they included CR, CRi, and PR. Patients with stable disease (SD), progression or not evaluable after 3 or 6 cycles were considered as no responders.

### Quantification of BCL2L10 positive cells

Flow cytometry was performed on fresh BMMC obtained before AZA onset, and after 3 or 6 cycles of treatment. BMMC were isolated by density centrifugation (Ficoll-Paque Plus). Cells were fixed by paraformaldehyde 3%, permeabilized with Triton 0,1%, incubated with an anti-human BCL2L10 rabbit antibody (Cell Signaling Technology, Beverly, MA, USA) followed by secondary donkey anti-rabbit FITC-labeled antibody and tested with a Canto Becton Dickinson flow cytometer. Examples of flow plots are shown in Supplementary Figure 1.

### Statistical analysis

Continuous variables were described using median [Interquartile range] (minimum; maximum) and qualitative variables using counts and percentages. Non-continuous variables were compared using the chi-square test. Mann-withney and kruskall-wallis tests were used for continuous variables. The best cut off value for BCL2L10 was defined using spline terms in a Cox model. Overall survival (OS) and post FCM evaluation were calculated from AZA onset, and from the date of FCM evaluation, respectively, and assessed by the Kaplan-Meier method. Statistical analysis was performed with SPSS v.22 (SPSS Inc., Chicago, IL, USA).

## SUPPLEMENTARY MATERIALS FIGURES AND TABLES


