# Supplementary material for: BCL2L10 positive cells in bone marrow are an independent prognostic factor of azacitidine outcome in myelodysplastic syndrome and acute myeloid leukemia
Source: Oncotarget. 2017 Apr 27;8(29):47103–9. doi: 10.18632/oncotarget.17482 (PMC5564547; doi:10.18632/oncotarget.17482)
Supplement: Supplementary file 1 [file oncotarget-08-47103-s001.pdf]

BCL2L10 positive cells in bone marrow are an independent prognostic factor of azacitidine outcome in myelodysplastic syndrome and acute myeloid leukemia

Supplementary Materials

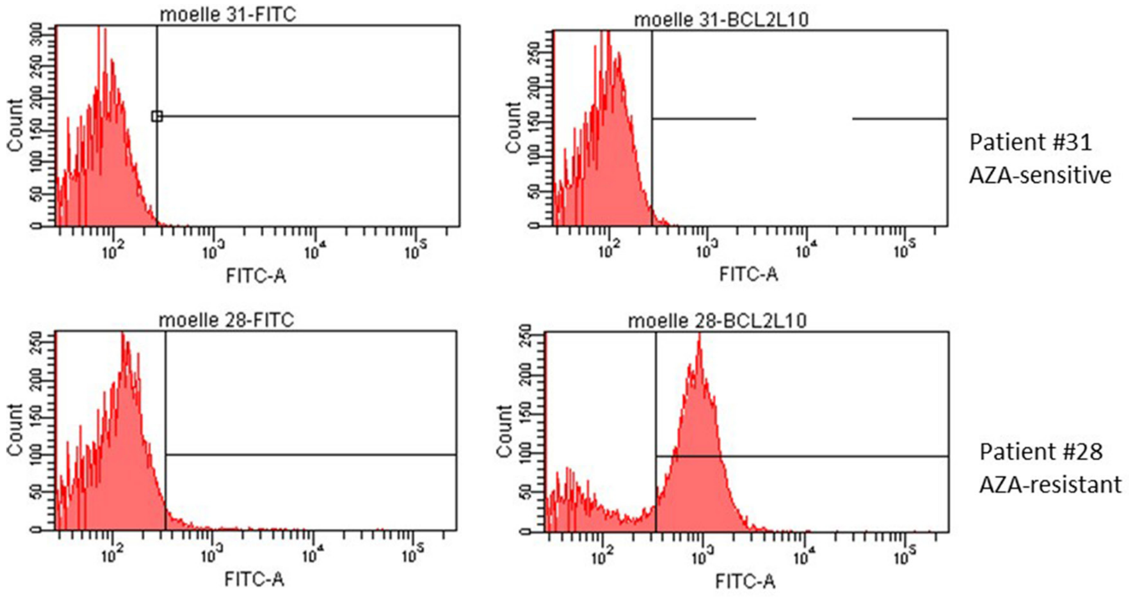

Supplementary Figure 1: Examples of BCL2L10 quantification by flow cytometry in AZA sensitive and resistant patients.

Supplementary Table 1: Characteristics of population depending on BCL2L10 percentage before AZA onset

	BCL2L10 before AZA onset		*p*-value
	< 10%	≥ 10%	
**WHO classification (%)**			0.07
RA/RCMD	44	21	
RAEB-1	16	5	
RAEB-2	20	26	
AML	20	48	
**Response (%)**			0.08
CR	14	0	< 0.0001 for CR/mCR
m-CR	36	0	
PR	0	20	
HI	21	0	
SD	29	20	
Progression	0	60	
**IPSS (MDS and AML ≤ 30%) (%)**			0.01
Low	8	13	
Int-1	56	7	
Int-2	17	40	
High	19	40	
**IPSS-R (MDS and AML ≤ 30%) (%)**			0.25
Very low	7	0	
Low	5	6	
Intermediate	37	11	
High	12	22	
Very high	39	61	
